# Trajectory-Based Air-Writing Recognition Using Deep Neural Network and Depth Sensor

**DOI:** 10.3390/s20020376

**Published:** 2020-01-09

**Authors:** Md. Shahinur Alam, Ki-Chul Kwon, Md. Ashraful Alam, Mohammed Y. Abbass, Shariar Md Imtiaz, Nam Kim

**Affiliations:** 1 Department of Computer and Communication Engineering, Chungbuk National University, Cheongju, Chungbuk 28644, Korea; shahinur@chungbuk.ac.kr (M.S.A.); kwon@osp.chungbuk.ac.kr (K.-C.K.); myehiaa@chungbuk.ac.kr (M.Y.A.); shariar@chungbuk.ac.kr (S.M.I.); 2 Department of Computer Science and Engineering, BRAC University, Dhaka 1212, Bangladesh; ashraful.alam@bracu.ac.bd

**Keywords:** air-writing, digit recognition, depth sensor, TOF camera, LSTM, CNN, trajectory recognition, human-computer interaction

## Abstract

Trajectory-based writing system refers to writing a linguistic character or word in free space by moving a finger, marker, or handheld device. It is widely applicable where traditional pen-up and pen-down writing systems are troublesome. Due to the simple writing style, it has a great advantage over the gesture-based system. However, it is a challenging task because of the non-uniform characters and different writing styles. In this research, we developed an air-writing recognition system using three-dimensional (3D) trajectories collected by a depth camera that tracks the fingertip. For better feature selection, the nearest neighbor and root point translation was used to normalize the trajectory. We employed the long short-term memory (LSTM) and a convolutional neural network (CNN) as a recognizer. The model was tested and verified by the self-collected dataset. To evaluate the robustness of our model, we also employed the 6D motion gesture (6DMG) alphanumeric character dataset and achieved 99.32% accuracy which is the highest to date. Hence, it verifies that the proposed model is invariant for digits and characters. Moreover, we publish a dataset containing 21,000 digits; which solves the lack of dataset in the current research.

## 1. Introduction

Writing in the air is a process to write something in a 3D space by using gestures or trajectory information. It allows users to write in a touchless system. Especially, it is useful when traditional writing is difficult such as gesture-based interaction, augmented reality (AR), virtual reality (VR), etc. Air-writing solves such issues. In pen-and-paper-based systems, characters are written in a multi-stroke manner. However, air-writing has no such option [1], which represents its principal drawback. The gesture-based writing is a helpful way to avoid this problem; nevertheless, it is not an optimum solution [2] due to the limitations of gesture varieties. Normally, the number of gestures is limited by human posture, but it is possible to increase the number of gestures by combining them [3]. However, remembering all of these is a little difficult for new users. On the other hand, users can write in the same order as traditional methods in the air-writing system.

Trajectory-based writing is performed in an imaginary box in front of a 2D [4,5] or 3D [6,7,8] camera. The principal problems when imposing a virtual boundary are spatiotemporal variability and segmentation ambiguity. It is difficult for even the same user to repeat the same trajectory again in the same position and pattern. Recently, Leap Motion and Kinect cameras have greatly advanced the emerging field of 3D vision-based trajectory tracking; it is now possible to write in the air without the need for any extra device or sensor. Some Kinect-based online handwriting recognition system has been developed [6,7,9,10]; this includes long-range body posture and finger tracking system. Leap Motion provides a precise finger location in the 3D space [11]. They used a heuristic approach to analyze the stroke length between two successive sequences. We prioritized usability when choosing a device. Kinect is a long-range tracking device and better for human pose or skeleton detection. Besides, Leap Motion is a small device that has a short-range but affords millimeter-level accuracy. This device is useful in a tabletop and VR environment. On the other hand, the Intel RealSense SR300 camera is an intermediate-level device that minimizes this tradeoff and provides an optimal solution for our purpose.

The trajectory-based writing system unveils new horizons of possibilities [6,7,12,13,14,15,16]. Trajectory estimation is more complicated than evaluation of pen-up/pen-down activity; users employ different writing styles and speeds, which in turn yield different trajectories. However, some state-of-the-art motion sensors and devices give us redemption from this problem. Gloves, wearable devices [12,14] and Wi-fi based [16] sensors yield accurate trajectory information, but for the general users they are hard to write with and to adapt to. The Leap Motion [11,17], Microsoft Kinect [6,7] and Intel RealSense cameras [18] provide effortless trajectory data. In this research, we used the Intel RealSense 3D depth camera to collect trajectory information.

It is essential to remove erroneous temporal and spatial trajectory data by applying some filters and normalization techniques. However, zigzag effects persist due to the special writing style. Users write digits in the 3D space inside an imaginary box, which is not fixed; no delimiter identifies the boundary of the region of interest. Hence, the trajectory is shaky whereas unwanted sequences are drawn that make the system more challenging. In that case, it needs to be normalized. Normalization is imperative to prune weak features. Machine learning-based approaches require subjective feature selection, which is a challenging task. Better features yield better results. However, the deep learning-based approach obviates the need for manual feature generation. Therefore, we implemented two efficient deep learning-based algorithms, named the long short-term memory recurrent neural network (LSTM) and the convolutional neural network (CNN). The LSTM network outperforms for all cases.

The main contributions of this paper are as follows.

(1) We design two efficient deep learning models allowing accurate air-writing digit recognition. The proposed LSTM and CNN networks are robust under different experimental conditions such as normalized/non-normalized situations. However, the 3D normalized trajectory provides a better result, especially for a distorted case.
(2) We create a large publicly available dataset that will play a vital role in deep learning and machine learning. The dataset contains 21,000 trajectories, which is sufficient for any deep learning algorithm. Among those, 1000 test trajectories are also included to measure the model’s performance in the unknown data.
(3) We verify the accuracy and feasibility of the model using our own dataset and publicly available dataset (6DMG). The quantitative performance comparison shows that the proposed model outperforms with the prior work.

The rest of the paper is organized as follows. In Section 2, we discuss the related prior work. The methodology is described in Section 3 including trajectory collection and network design. Section 4 and Section 5 are an experiment and result section, respectively. In the results section, we compare the model performance with a publicly available dataset. Section 6 contains conclusion and future work.

## 2. Related Work

Standalone devices [12,13,14,19,20,21,22] have been used for air-writing and gesture-based digit recognition. Some cost as little as $1 [19], are readily applicable and serve as a prototype for user interface (UI) designers who lack knowledge of pattern recognition. Performance has been compared among various algorithms and devices. The MEMS gyroscope [20], accelerometer [14,23,24], motion sensor [12], Wiimote [13], HP slate 2 [21], Wi-fi [16], and analog inertial measurement unit [22,25] are the leading devices and sensors for trajectory estimation in a 3D spatiotemporal domain. Wi-fi based writing system is new in this field, authors named it Wri-Fi and utilized the channel state information which derived from wireless signals to experience the device-free air writing system. Recently, inertial sensor-based portable devices have been used to collect human motion trajectory information from accelerations [14,20,22]. All current devices are handheld or attached to the body. Importantly, the sensors operate without external control. Researchers have focused on reducing the error from trajectory by manipulating the velocities and signals of inertial sensors [25,26]. Many of them have been focused on designing efficient and effective algorithms to minimize the tradeoff between complexity and accuracy. Motion sensing technologies ascribe some behavioral issues. For example, wearing a sensing device is often considered burdensome. The gyroscope-based method uses the extended Kalman filter to detect the motion appropriately. The main purpose of that method was to use the system on a digital pen, and the accelerometer-based devices are used for the same purpose [14]. This pen features a triaxial accelerometer with a wireless transmitter. It is applicable for both writing and hand gesture detection. The IMU pen [25] and Magic Wand [27] are inertia sensor-based input devices. The Wiimote and HP Slate are commercial sensor-based motion detection devices. Recent studies used a six-degree-of-freedom (6DMG) dataset [13,15] collected by the Wiimote device. The word-based recognition error rate was reasonably low. Moreover, an extensive comparison has been done between air-writing and virtual keyboard. In terms of accuracy and ease, air-writing was more accurate and simpler than the virtual keyboard. However, these sensors are handheld, which makes them difficult and complicated to use. Therefore, we developed a 3D vision-based approach and used a depth sensor-based camera known as the time of flight (TOF) camera whose main working principle is the IR sensor. Since the TOF camera measures the distance between the objects and camera, which is calculated using the reflection principle, it can work in a low-light environment.

There are different algorithms that have been used for air-writing recognition. The most prominent algorithms are hidden Markov model (HMM) [8,11,13,16,22], dynamic time warping (DTW) [6,7,28], support vector machine (SVM) [7,22], and bi-directional LSTM (BLSTM) [29]. The HMM is a Markov model containing hidden states that play an important role. DTW is the time series analysis algorithm measuring the similarities between pairs of temporal sequences. HMM, DTW, and SVM based recognizers assume that the observation is conditionally exclusive. Therefore, overlapping or long-range observable feature processing is complex and sometimes erroneous [6]. Discriminative methods avoid this problem because they accommodate nonlocal dependencies. However, HMM is the widely used algorithm in the field of gesture recognition and air-writing. Nowadays, LSTM has become popular for time series prediction, speech recognition, handwriting recognition, etc.; hence, we used the LSTM algorithm for this research. Nowadays CNN is also becoming popular for different applications for its different variants and robustness. For this research, we also used a depth-wise CNN network.

Recently, researchers have been following different strategies for air-writing recognition. Nguyen and C. Bartha introduced shape writing and compared it with the Swype on a smartphone [21]. It is shown that shape writing performs better as a virtual keyboard. Amma et al. proposed an air-writing system using the inertial measurement unit, which required attaching with the hand [22]. The HMM recognizer and SVM classifier were used to detect the character in the air. The main drawback of this work is that it is handheld; always attaching this device with the hand is very difficult and tedious. To overcome this situation, Qu et al. proposed a Kinect based online digit recognition system using DTW and SVM [7]. Lately, Mohammadi and Maleki proposed a Kinect based Persian number recognition system. However, those are not full writing systems. Chen et al. [17] and Kumar et al. [11] proposed a full writing system in the air including character and word. Both used Leap Motion as a trajectory detection device and HMM as a recognizer, but the accuracies were not significant. However, Kumar et al. used the BLSTM algorithm and showed that the accuracy was higher than HMM. Most of the research has been done by using the trajectory information directly, i.e., using the temporal information. On the other hand, Setiawan and Pulungan [30] proposed a 2D mapping approach, in which trajectories were collected by the Leap Motion device and converted to the 2D image matrix like the popular MNIST dataset. Nowadays, WiFi and Radar-based technology have become popular. Fu et al. [16] proposed a Wi-Fi device-based (named as Wri-Fi) method using principal component analysis (PCA) and HMM algorithm. The main drawback of this work is that accuracy is not reasonable. However, Arsalan and Santra [29] solved the accuracy issues using millimeter-wave radar technology. They used three radars calibrated with trilateration techniques to detect and localize the hand marker, which is troublesome to implement for real-life applications. Therefore, we were motivated to develop a vision-based and hassle-free system for all users. At the same time, we achieved very good recognition accuracy.

## 3. Methodology

**Figure 1:**
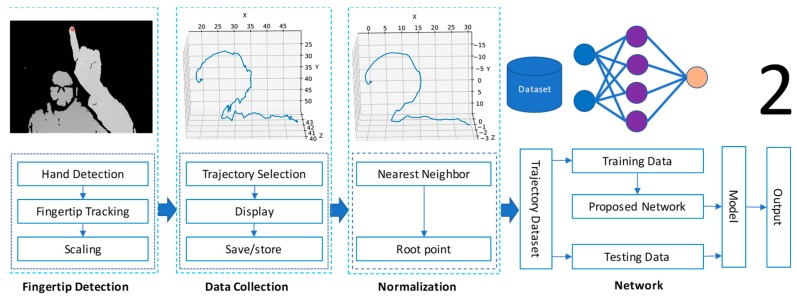
Block diagram for the proposed method including the fingertip detection, data collection, normalization, and network design.

The whole process is divided into four principal parts: fingertip detection, data collection, normalization, and network design. A complete block diagram is shown in Figure 1 to describe the details of the proposed method. The following subsections provide an in-depth explanation for each part.

### 3.1. Fingertip Detection

Fingertip detection was performed by an Intel RealSense SR300 camera. It is a widely used TOF camera for gesture detection, finger joint tracking, and depth-sensing research area. Firstly, hand segmentation and detection were done. Normally, there are 22 finger joints in our hands. Amongst them, the index fingertip was tracked for trajectory writing for user convenience. However, the trajectory was drawn through a virtual window. To fit and display in the physical window (such as a computer screen), scaling was required. We calculated the physical distance by multiplying the window size and the adaptive value for both x (horizontal) and y (vertical) directions. The adaptive value is the normalized value between 0 and 1 collected through the RealSense camera. In the User Interface (UI), the window sizes were 640 and 480 for x and y, respectively. The original pixel value was calculated by multiplying the window size and adaptive value.

### 3.2. Data Collection

**Figure 2:**
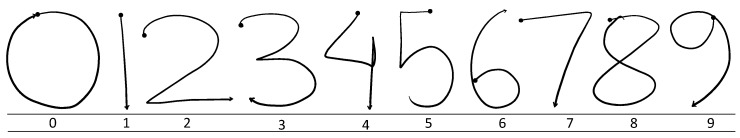
The digit writing order. The dot and the arrow symbol represent the starting and ending point, respectively.

A simple interactive UI was designed requiring minimal instructions. 10 participants (8 males and 2 females) aged 23 to 30 years were recruited; all were graduate students. The writing order is shown in Figure 2, which is similar to the traditional writing order. We did not apply graffiti or uni-stroke writing constraints [31]. The stroke orders for each letter were defined in the usual manner, i.e., the box-writing process [13]. There are two types of approaches to recognize the air-writing system, online and offline. Herein, we followed an offline method which is also known as the ‘push-to-write’ approach to collect the data [11,13,28,32]. In this process, users were requested to write digits in front of the depth camera, and that was collected as a spatial trajectory sequence.

The collected RealSense trajectory digits (RTD) dataset parameters are listed in Table 1. The digits 4, 5, and 8 are relatively longer than the other trajectories. Digit 1 had the shortest and 4 had the longest trajectory. Depending on personal preference, each user could employ a very small or very large writing area. However, the differences between the maximum and minimum length of the same trajectory are large, showing that the dataset contains a variety of data points. The mean Equation (1) and standard deviation (STD) indicate that most data points are distributed within a reasonable range. The variance Equation (2) indicates the spread of the dataset; this is helpful to design the input layer of the deep learning algorithm. The STD Equation (3) represents the deviation from the mean value. Although the minimum and the maximum ranges are numerous, the minimal STD difference indicates that most of the data points reside within the normal range.

(1) $$\bar{x}=\frac{1}{n}\sum_{i=1}^{n}x_{i}$$

(2) $$\sigma^{2}=\frac{1}{n-1}\sum_{i=1}^{n}{\left(x_{i}-\bar{x}\right)}^{2}$$

(3) $$\sigma =\sqrt{\frac{1}{n-1}\sum_{i=1}^{n}{\left(x_{i}-\bar{x}\right)}^{2}}$$

where $\bar{x}$ is the mean value, $x_{i}$ is the ith datum, $\sigma^{2}$ is the variance, and σ is the STD. Some data were collected for testing purposes to verify the accuracy of unknown data.

### 3.3. Normalization

The main challenge for air-writing is that the trajectory is zigzag, i.e., not smooth, thus requiring normalization before feeding the network. We employed two normalization techniques—the nearest neighbor and root point. The details are as follows:

#### 3.3.1. Nearest Neighbor Point Normalization

The nearest neighbor point normalization technique is simple and heuristic. As it is not a pen-up/pen-down system, some displaced points are captured, which may change the trajectory shape. To deal with this situation, averaging the nearest-point transformation is used to change the deviated line to a smooth and straight line. The Equations (4) to (6) are applied to calculate the nearest point.

(4) $$x_{i}=\frac{1}{n}\sum_{i=0}^{n}x_{i}$$

(5) $$y_{i}=\frac{1}{n}\sum_{i=0}^{n}y_{i}$$

(6) $$z_{i}=\frac{1}{n}\sum_{i=0}^{n}z_{i}$$

Here *i* is the individual position in a trajectory and *n* is the number of points considered during normalization. It is observed from the experiment that considering six points are optimal. More points smoothened the trajectory, but it shrank the corner(s) that produce distorted shape. On the other hand, lower points could not properly normalize the trajectory.

#### 3.3.2. Root Point Normalization

**Figure 3:**
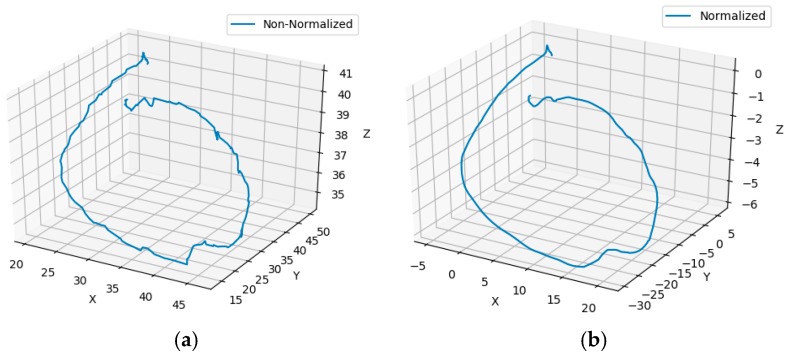
Three-dimensional trajectory visualization: (**a**) sample trajectory before normalization and (**b**) trajectory after normalization.

During air-writing, users write in an imaginary (“virtual”) box in the air. All digits are written in the first quadrant in a cartesian plane but in a different position. The virtual box does not have fix boundaries or margins; thus, the same digit may be written in different positions, even by the same user. This causes a random initial position. We used root point translation to generalize the starting point. By doing this, all the starting points commenced from the root coordinate. The Equations (7) to (9) calculate the root point.

(7) $$x_{i}=x_{0}-x_{i}$$

(8) $$y_{i}=y_{0}-y_{i}$$

(9) $$z_{i}=z_{0}-z_{i}$$

where [$x_{0}$, $y_{0}$, $z_{0}$] is the starting point of a sequence and [$x_{i}$, $y_{i}$, $z_{i}$] is the instantaneous point derived from the entire trajectory. The non-normalized digit 0 is shown in Figure 3a; it is noisy and has a zigzag effect, while the fully normalized trajectory (Figure 3b) is smooth. In this figure, the *x* and *y* are the distance value in the virtual window; i.e., the passing distance between the start and end position. The *z* is the distance between the hand fingertip and the camera. All are in centimeters (cm). The negative value in Figure 3b indicates the relative distance. It is due to using the Equations (7) to (9) in the normalization process. The geometric root point is considered as the starting point; for example, −5 in the *x*-direction, −30 in the *y*-direction, and −6 in the *z*-direction indicate that the point is 5 cm left, 30 cm below, and 6 cm closer to the camera from the start position, respectively. Here, the negative sign indicates the direction, not the mathematical operator.

### 3.4. The Dataset

In our work, two datasets are employed, one is self-collected (RTD) and another is the 6DMG [33]. The details are described below.

#### 3.4.1. RTD Dataset

RTD is our self-collected trajectory dataset which contains 20,000 trajectories, i.e., 2000 data points for each digit. The writing order and parameters are shown in Figure 2 and Table 1, respectively. To the best of our knowledge, this is the largest digit dataset currently available based on the number of trajectories per digit. The RTD dataset is freely available on the internet as are detailed instructions for use. The RTD dataset: https://www.shahinur.com/en/RTD/.

#### 3.4.2. 6DMG

**Figure 4:**
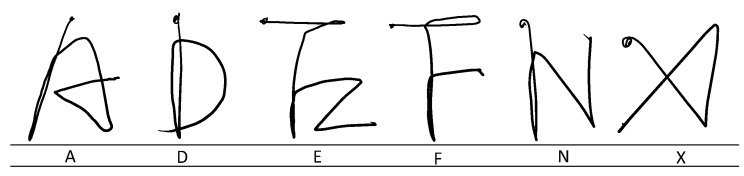
Sample character from 6DMG dataset.

6DMG is a motion gesture dataset containing gestures and alphanumeric air-writing data [33]. The dataset was collected through the Wiimote device, performed by 28 participants. Wiimote is a handheld device that works based on acceleration and angular speed. The orientation of this device was computed using the inertial measurement. In this study, we employed this 6DMG air-writing dataset. Some sample characters are shown in Figure 4. All characters are written in a uni-stroke manner.

### 3.5. Network Design

In this research, we employed two state-of-the-art neural network algorithms, CNN and LSTM. CNN and LSTM work based on convolution and recurrent units, respectively. Both are widely applicable for time series prediction.

#### 3.5.1. LSTM Network

**Figure 5:**
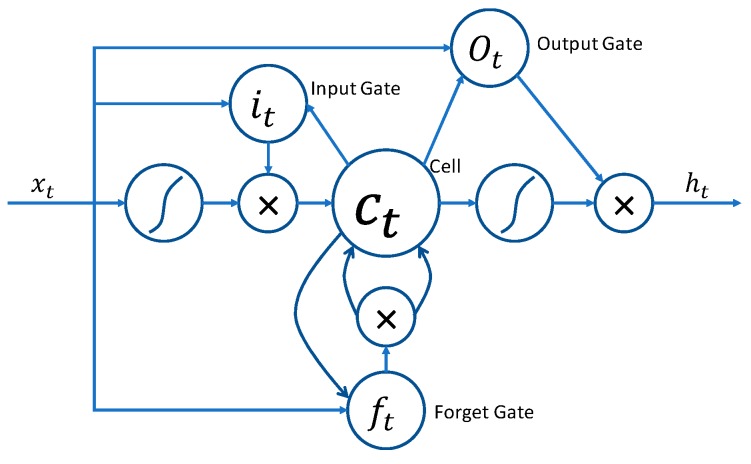
A Single long short-term memory (LSTM) unit. Here, $c_{t}$, $i_{t}$, $f_{t},$ and $o_{t}$ represents cell, input gate, forget gate, and output gate, respectively.

LSTM [34] is a variant of a recurrent neural network (RNN). Unlike the standard feed-forward network, LSTM has feedback connections that make it significant. The architecture of an LSTM cell is shown in Figure 5.

The LSTM layer is composed of a cell containing an input Equation (10), forget Equation (11), and output Equation (12) gates. It is also known as a cell state Equation (13). This state is modified by the forget gate. It helps to decide what to be removed from the previous state, and thus, the cell keeps only the expected information. The output gate in a cell passes the information to the next state.

(10) $$i_{t}=\sigma \left(W_{i}x_{t}+U_{i}h_{t-1}+b_{i}\right)$$

(11) $$f_{t}=\sigma \left(W_{f}x_{t}+U_{f}h_{t-1}+b_{f}\right)$$

(12) $$o_{t}\;=\;\sigma \left(W_{o}x_{t}+U_{o}h_{t-1}+b_{o}\right)$$

(13) $$c_{t}=f_{t}\circ c_{t-1}+i_{t}\;⊙\;\sigma \left(W_{c}x_{t}+U_{c}h_{t-1}+b_{c}\right)$$

where *i*, *f*, and *o* are the input, forget, and output gate, respectively. The weight matrices *W* and bias *b* is the parameter which is required to learn during the training. The symbol ⊙ is the Hadamard product [35] which produces the same dimensional matrix as the input.

The proposed network contains two LSTM and two dense layers. The input layer is the very first layer in the network, and it does not compute anything; instead, it transfers inputs to the next LSTM layer. The trajectory length is not fixed. Each trajectory has a different 3D spatial points, and even the same digit may vary in length depending on the writing speed and direction. However, fixed-length sequences are required to feed the LSTM-RNN. Therefore, the input layer is set to the maximum trajectory length.

**Figure 6:**
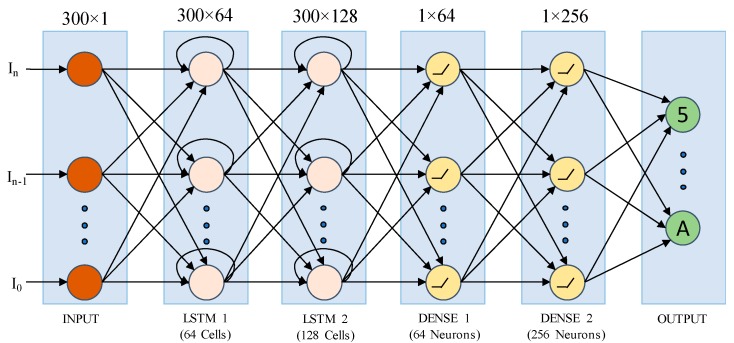
Network diagram of the proposed LSTM network indicating the input, output and hidden layers. Here $I_{0}\;\mathrm{and}\;I_{n}$ is the first and last point for an individual trajectory, respectively. There are two LSTM and dense layer with multiple neurons. The output layer is the result of the model.

The proposed LSTM model has been shown in Figure 6; $I_{0}$, $I_{n}$ are inputs containing the *x*, *y*, and *z* values from the trajectory. LSTM 1 and LSTM 2 contain 64 and 128 cells, respectively. The cell is used to compute the output activation of the LSTM unit. To employ the cross-validation, we used a mini-batch size of 256. DENSE 1 and DENSE 2 contain 64 and 256 neurons, respectively. DENSE 1 and 2 employ the Rectified Linear Unit (ReLU) activation function Equation (14), which is simple and easy to use. The most important property of ReLU is that the output is straightforward, and it does not saturate. The ReLU function eliminates the negative part of the output by transmitting only the positive part to the next layer.

(14) f(x)=max(0,x)

where *x* is the input to the neuron. However, the softmax activation function Equation (15) is used in the output layer. This yields an integral output whereas the output of a SoftMax function is a probability between 0 and 1.

(15) $$\sigma {\left(z\right)}_{j}=\frac{e^{Z_{j}}}{\sum_{k=1}^{K}e^{Z_{j}}}$$

Dropout is a regularization technique patented by Google for reducing overfitting in neural networks by preventing complex co-adaptations in training data. It helps to prevent overfitting [36] by randomly subsampling the data and is used in the dense layer. The dropout rate is 0.5. Moreover, an extension of the stochastic gradient descent algorithm Adam [37] is employed to optimize the model at a learning rate of 0.0005. Its implementation is straightforward and computationally efficient requiring a little memory.

#### 3.5.2. CNN Network

A CNN network consists of an input and output layers composed of multiple hidden layers. Hidden layers include convolution, pooling, and activation layers. CNN has different variants based on the application and dataset. The normal CNN network contains lots of calculations due to the convolution and pooling layer. Hence, we applied the separable convolution layer which is faster than the general CNN layer. It is widely known as a depth wise convolution.

**Figure 7:**
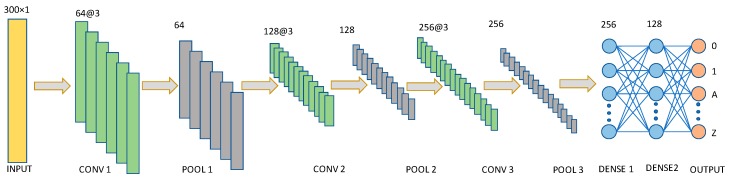
Proposed convolutional neural network (CNN) model. The model has three convolutions, three max-pooling and 2 dense layers.

The proposed CNN network is shown in Figure 7. Like the LSTM network, the input layer is also set to the maximum length of the trajectories. The input layer is a 300 × 1-dimensional vector that transfers the input value to the 1st separable convolution layer. Convolution layers 1, 2, and 3 contain 64, 128, and 256 channels, respectively. In all the cases the filter size is 3. Each convolution layer is associated with the 1D max-pooling layer. There are two dense layers containing 256 and 128 neurons, respectively. Dropout rate 0.5 is used in the DENSE1 and DENSE2 layers. The ReLU Equation (14) activation function is used for all the cases except the output layer. The softmax Equation (15) regularization is used in the output layer. Adam is used as an optimizer with a learning rate of 0.0001 and categorical cross-entropy as a loss function.

## 4. Experimental Setup

The experiment was done by connecting an Intel RealSense SR300 camera with a computer. A graphical user interface (GUI) was designed to collect the appropriate trajectory data. The C# and Python programming languages were used for interfacing and training, respectively. The proposed networks were implemented in Keras high-level API over the TensorFlow backend.

**Figure 8:**
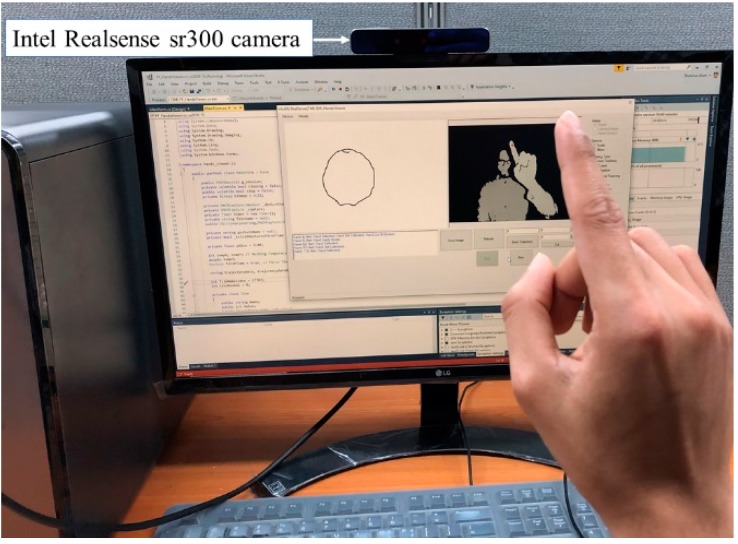
Experimental setup for air-writing dataset collection interface.

**Figure 9:**
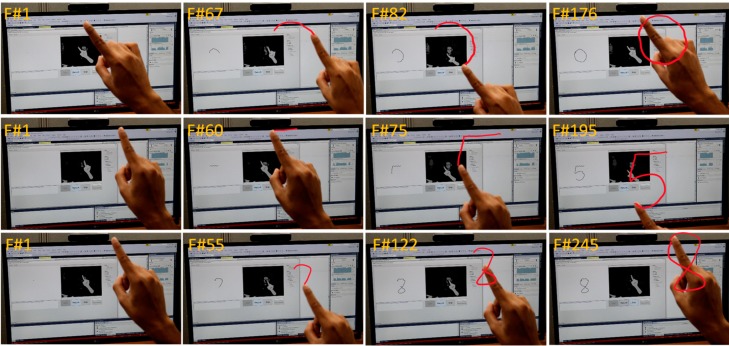
Sample digit writing order, frame by frame representation of digits 0, 5, and 8.

The trajectory was captured in real-time (50 fps). We used the NVIDIA GeForce GTX 1050 Ti graphics processing (GPU) unit with 32 GB memory to speed up the training process. The experimental environment is shown in Figure 8. The digit 0 was written in the air and tracked by the RealSense camera. It is not normalized; hence, the trajectory is a little bit distorted. Some of the complex digits and corresponding sample frames are shown in Figure 9. The frame by frame representation helps to understand the motion for the digit writing order. The trajectory capturing process started from the first frame (F#1). However, the ending frame is different for each individual character due to the different motion and writing patterns.

### 4.1. User Interface

In the user interface, there were three basic buttons to start, stop, and save trajectory. The GUI displays both depth information and the trajectory. The depth and the trajectory part were used to display the full hand indicating the tracked fingertip point and the captured trajectory, respectively. The GUI was very simple, so minimal instruction was needed. It was also highly interactive, featuring real-time trajectory capture and display. For simplicity, each digit was shown in 2D cartesian coordinates, but it is three dimensional. In Figure 8, digit 0 is shown on the left side; the depth maps of the finger and body appear on the right. As soon as the ‘start’ button clicked, the camera became active and started to detect the fingertip position. If the drawn trajectory is the expected trajectory, the user clicks the ‘stop’ button. If the trajectory is not expected or the user cannot draw properly, then they can click the ‘Refresh’ button to clear the window. Finally, the trajectory can be saved by pressing the ‘Save’ button. Users do not need to write down the label, the system itself incrementally generates one by one. Basically, the ‘start’ and ‘stop’ buttons in the UI control the initial and end points, respectively. To trim the unwanted starting or ending points, we used the ‘cut’ button to eliminate the terminal point according to the instructions.

### 4.2. Usability

A user study was conducted to verify usability. Users were directed to write in the air in front of the camera. Initially, they needed training, but that is for a short time. The average training time was less than 3 min. In the first 5–10 attempts, they were experiencing a few problems such as shaky effect, stiff and numb hand, etc., but later it was fixed. Most users gave positive feedback and appreciated this work.

## 5. Results and Discussions

### 5.1. Parameter Tuning

The model was trained several times; the dataset contained 20,000 trajectories and 10-fold cross-validation was employed. Thus, each iteration contained 18,000 training and 2000 validation data. We tried different batch sizes and iterations to tune up the parameters.

**Figure 10:**
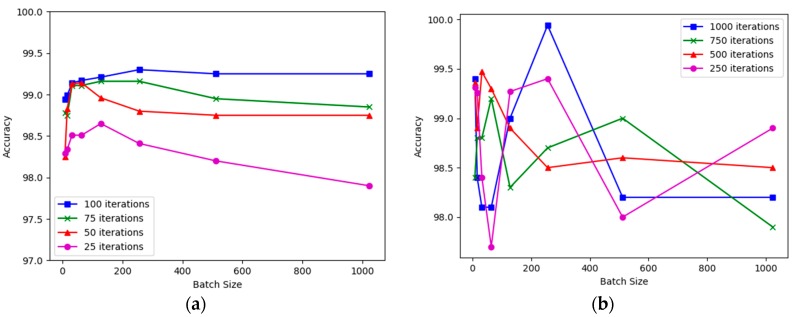
Parameter tuning: (**a**) batch size vs accuracy for LSTM network, (**b**) batch size vs accuracy for CNN network. The convergence is faster in LSTM rather than CNN in terms of the number of iterations.

The LSTM model ran for 25, 50, 75, and 100 times. It is shown in Figure 10a that the accuracy is different for each iteration and batch size. As depicted in the figure, the highest accuracy was found in 256 batch size for 100 iterations. Therefore, for LSTM, the considerable batch size and iterations are 256 and 100, respectively.

Unlike LSTM, the CNN model ran for 250, 500, 750, and 1000 times (shown in Figure 10b). It requires higher iterations than LSTM to converge. The highest and optimum accuracy was found in 256 batch size with 1000 iterations. Hence, the rest of the discussion will be based on the 256 batch size, and 100 and 1000 iterations for LSTM and CNN, respectively. Datapoint in Figure 10 looks orderless because there is no definite rule that the accuracy will be linear respective to the iteration and batch size.

### 5.2. Evaluation of the Proposed LSTM and CNN Network

**Figure A1:**
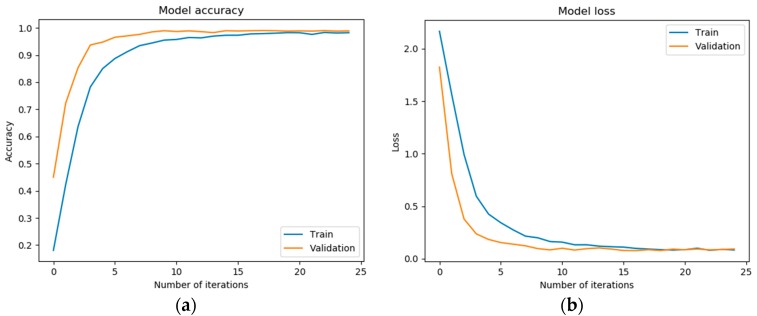
Model accuracy and loss for LSTM network: (**a**) accuracy over 100 iterations. (**b**) loss over 100 iterations.

**Figure A2:**
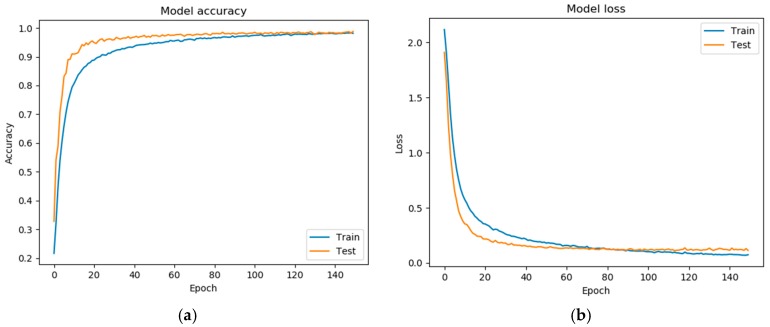
Model accuracy and loss for CNN network: (**a**) accuracy over 100 iterations, (**b**) loss over 100 iterations.

We performed an extensive experiment for the RTD dataset using LSTM and CNN networks. It was observed that the LSTM model achieves reasonable accuracy at around 20 iterations, after that it saturates. The accuracy over iteration is plotted in Figure A1 (Appendix A). Notably, a high peak was attained within 15 or 20 iterations. On the other hand, the CNN model saturates at 150 iterations (shown in Figure A2 (Appendix A)). Such sudden changes in model loss and accuracy indicate that the model adapts rapidly to the dataset.

Both CNN and LSTM models performed well. However, the highest accuracy was found for a fully normalized RTD dataset with LSTM. The details are shown in Table 2. The worst-case was found for the non-normalized data for both LSTM and CNN. It was also observed that the nearest neighbor was more important than the root point normalization technique.

**Figure A3:**
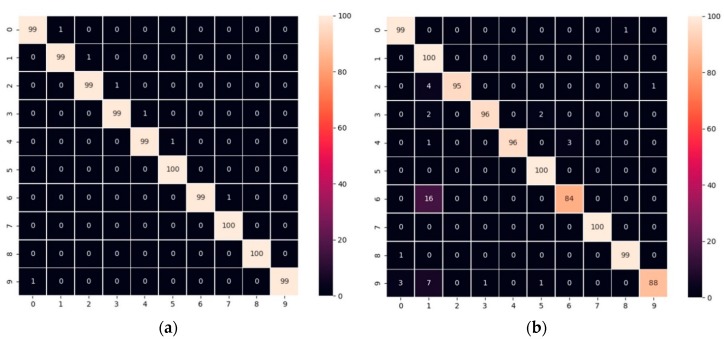
Confusion matrix for RTD dataset using: (**a**) LSTM and (**b**) CNN networks. The false recognition rate for CNN is higher than for LSTM.

The confusion matrix is shown in Figure A3 (Appendix A). for both CNN and LSTM models. The false recognition rate is higher for CNN. The digit 6 is recognized 16 times as 1 for CNN but it is solved automatically for LSTM. Compared to CNN, LSTM performed better in all cases. This is because of the time-series data as the trajectory has long term dependency and LSTM is especially capable of handling such kinds of issues. However, both CNN and LSTM are reasonable as the accuracy is 99.06% and 99.17%, respectively.

### 5.3. 6DMG Dataset Evaluation

The analysis for RTD dataset mentioned in the previous section just provides an overview of the proposed model but not exactly how good or bad the model is (because RTD has no benchmark yet.). That is why we used the 6DMG dataset to compare our proposed network side by side. The detailed comparison is shown in Table 3. Chen et al. performed an extensive analysis of this dataset [38]. They considered both user-dependent and user-independent cases; an explicit and implicit motion was also considered. The explicit motion performed better than the implicit. Hence, the accuracy was 96.9% for explicit motion character with an HMM classifier. Later they improved their model and achieved 99.2% accuracy which is higher than before [13]. Xu and Xue followed deep learning (LSTM) based approach but the accuracy is less than the previous work [32]. In that contemporary time, Yana and Onoye combined the BLSTM and CNN features and achieved 99.27% maximum accuracy [39].

In this case, the proposed LSTM network outperforms with the previous work and found 99.32% accuracy which is the highest to date. In this case, the accuracy of the proposed CNN model is similar to [39]. Previous researchers have also used the LSTM layer; however, we used two consecutive LSTM layers and normalized the dataset before feeding the network; hence, the accuracy is higher than others.

## 6. Conclusions

In this paper, we proposed a trajectory-based air writing recognition system using an Intel RealSense SR300 camera and developed two deep learning-based algorithms to recognize the trajectory. This is a paperless writing system. Researchers previously used different motion sensors and handheld devices; instead, we used a vision-based approach for better user experience. To verify the method and assess its accuracy, we proposed two neural network models—LSTM and CNN.

We thoroughly assessed the method using different normalization conditions and found that the normalized 3D data were optimal. We also employed the 6DMG dataset and achieved the highest accuracy. The main contribution of this paper is to design a network for higher recognition accuracy and solve the dataset issues in the current research. The highest recognition accuracies for RTD and 6DMG datasets for CNN are 99.06% and 99.26%, respectively. However, the highest recognition accuracy was found for LSTM, which is 99.17% and 99.32% for RTD and 6DMG datasets, respectively. This can be achieved within a reasonable iteration, which proves that the model is very efficient and learns very fast. We performed a comparative analysis of our work with prior research and observed that the accuracy is relatively higher. In the future, we will try to design and develop a model for continuous writing systems to apply to AR/VR or gesture alternatives.

## Figures and Tables

**Table 1:** Parameter of the RealSense trajectory digits (RTD) trajectory dataset.

Digit	Maximum	Minimum	Mean	Variance	STD
0	224	26	60	619	24
1	144	13	36	232	15
2	228	26	73	676	26
3	218	35	74	614	24
4	255	39	90	729	27
5	265	37	80	613	24
6	235	25	58	414	20
7	155	21	53	314	17
8	250	30	80	589	24
9	223	22	64	482	21

**Table 2:** Performance Evaluation for LSTM and CNN Model.

Algorithm	Accuracy (%)
CNN + Without Normalization	98.26
CNN + Nearest Neighbor	98.89
CNN + Root Point	98.73
CNN + Nearest Neighbor + Root Point	99.06
LSTM + Without Normalization	98.68
LSTM + Nearest Neighbor	99.08
LSTM + Root Point	99.02
LSTM + Nearest Neighbor + Root Point	99.17

**Table 3:** The proposed LSTM and CNN model evaluation using the 6DMG dataset.

Algorithm	Algorithm	Accuracy (%)
[38]	HMM	96.9
[13]	HMM	99.2
[32]	LSTM	93.98
[39]	BLSTM	99.27
Proposed Method	CNN	99.26
	LSTM	99.32

## Appendix A

The iteration versus accuracy and loss are plotted here for both LSTM and CNN networks. The networks converge very fast in terms of iterations.

The confusion matrix for LSTM and CNN network. It shows that the accuracy is a little higher in LSTM than CNN. However, the difference is not significant.

## References

[1] Welch G., Foxlin E. (2002). Motion tracking: No silver bullet, but a respectable arsenal. IEEE Comput. Graph. Appl..

[2] Liu H., Wang L. (2018). Gesture recognition for human-robot collaboration: A review. Int. J. Ind. Ergon..

[3] Modanwal G., Sarawadekar K. (2016). Towards hand gesture based writing support system for blinds. Pattern Recognit..

[4] Frolova D., Stern H., Berman S. (2013). Most Probable Longest Common Subsequence for Recognition of Gesture Character Input. IEEE Trans. Cybern..

[5] De O., Deb P., Mukherjee S., Nandy S., Chakraborty T., Saha S. Computer vision based framework for digit recognition by hand gesture analysis. Proceedings of the 2016 IEEE 7th Annual Information Technology, Electronics and Mobile Communication Conference.

[6] Poularakis S., Katsavounidis I. (2016). Low-Complexity Hand Gesture Recognition System for Continuous Streams of Digits and Letters. IEEE Trans. Cybern..

[7] Qu C., Zhang D., Tian J. (2015). Online Kinect Handwritten Digit Recognition Based on Dynamic Time Warping and Support Vector Machine. J. Inf. Comput. Sci..

[8] Mohammadi S., Maleki R. (2019). Air-writing recognition system for Persian numbers with a novel classifier. arXiv.

[9] Tian J., Qu C., Xu W., Wang S. KinWrite: Handwriting-Based Authentication Using Kinect. Proceedings of the 20th Annual Network and Distributed System Security Symposium.

[10] Zhang X., Ye Z., Jin L., Feng Z., Xu S. (2013). A new writing experience: Finger writing in the air using a kinect sensor. IEEE Multimed..

[11] Kumar P., Saini R., Roy P.P., Dogra D.P. (2017). Study of Text Segmentation and Recognition Using Leap Motion Sensor. IEEE Sens. J..

[12] Zhou Y., Dai Z., Jing L. A controlled experiment between two methods on ten-digits air writing. Proceedings of the 2016 16th IEEE International Conference on Computer and Information Technology.

[13] Chen M., AlRegib G., Juang B.-H. (2016). Air-Writing Recognition—Part I: Modeling and Recognition of Characters, Words, and Connecting Motions. IEEE Trans. Hum.-Mach. Syst..

[14] Wang J.S., Chuang F.C. (2012). An accelerometer-based digital pen with a trajectory recognition algorithm for handwritten digit and gesture recognition. IEEE Trans. Ind. Electron..

[15] Yana B., Onoye T. (2018). Air-writing recognition based on fusion network for learning spatial and temporal features. IEICE Trans. Fundam. Electron. Commun. Comput. Sci..

[16] Fu Z., Xu J., Zhu Z., Liu A.X., Sun X. (2019). Writing in the Air with WiFi Signals for Virtual Reality Devices. IEEE Trans. Mob. Comput..

[17] Chen M., AlRegib G., Juang B.H. (2016). Air-Writing Recognition—Part II: Detection and Recognition of Writing Activity in Continuous Stream of Motion Data. IEEE Trans. Human-Machine Syst..

[18] Tordesillas J., Lopez B.T., How J.P. FaSTraP: Fast and Safe Trajectory Planner for Flights in Unknown Environments. Proceedings of the International Conference on Intelligent Robots and Systems.

[19] Wobbrock J.O., Wilson A.D., Li Y. (2007). Gestures without libraries, toolkits or training: A $1 recognizer for user interface prototypes. Proceedings of the 20th Annual ACM Symposium on User Interface Software and Technology—UIST ’07.

[20] Luo Y., Tsang C.C., Zhang G., Dong Z., Shi G., Kwok S.Y., Li W.J., Leong P.H.W., Wong M.Y. An Attitude Compensation Technique for a MEMS Motion Sensor Based Digital Writing Instrument. Proceedings of the 2006 1st IEEE International Conference on Nano/Micro Engineered and Molecular Systems.

[21] Nguyen H., Bartha M.C. Shape writing on tablets: Better performance or better experience?. Proceedings of the Human Factors and Ergonomics Society.

[22] Amma C., Georgi M., Schultz T. (2014). Airwriting: A wearable handwriting recognition system. Pers. Ubiquitous Comput..

[23] Kratz S., Rohs M. (2011). Protractor3D. Proceedings of the 15th International Conference on Intelligent User Interfaces—IUI ’11.

[24] Guerra-Casanova J., Avila C.S., Bailador G., de-Santos-Sierra A. Time series distances measures to analyze in-air signatures to authenticate users on mobile phones. Proceedings of the 2011 Carnahan Conference on Security Technology.

[25] Wang J.-S., Hsu Y.-L., Liu J.-N. (2010). An Inertial-Measurement-Unit-Based Pen With a Trajectory Reconstruction Algorithm and Its Applications. IEEE Trans. Ind. Electron..

[26] Hsu Y.L., Chu C.L., Tsai Y.J., Wang J.S. (2015). An inertial pen with dynamic time warping recognizer for handwriting and gesture recognition. IEEE Sens. J..

[27] Cho S.J., Oh J.K., Bang W.C., Chang W., Choi E., Jing Y., Cho J., Kim D.Y. Magic Wand: A Hand-Drawn Gesture Input Device in 3-D Space with Inertial Sensors. Proceedings of the Ninth International Workshop on Frontiers in Handwriting Recognition.

[28] Chiu L.-W., Hsieh J.-W., Lai C.-R., Chiang H.-F., Cheng S.-C., Fan K.-C. Smart Multimedia. Proceedings of the Person Authentication by Air-Writing Using 3D Sensor and Time Order Stroke Context.

[29] Arsalan M., Santra A. (2019). Character Recognition in Air-Writing Based on Network of Radars For Human-Machine Interface. IEEE Sens. J..

[30] Setiawan A., Pulungan R. (2018). Deep Belief Networks for Recognizing Handwriting Captured by Leap Motion Controller. Int. J. Electr. Comput. Eng..

[31] Castellucci S.J., MacKenzie I.S. (2008). Graffiti vs. unistrokes. Proceedings of the Twenty-Sixth Annual CHI Conference on Human Factors in Computing Systems—CHI ’08.

[32] Xu S., Xue Y. A Long Term Memory Recognition Framework on Multi-Complexity Motion Gestures. Proceedings of the 2017 14th IAPR International Conference on Document Analysis and Recognition (ICDAR).

[33] Chen M., Alregib G., Juang B.H. 6DMG: A new 6D motion gesture database. Proceedings of the 3rd Multimedia Systems Conference.

[34] Hochreiter S., Schmidhuber J. (1997). Long Short-Term Memory. Neural Comput..

[35] Davis C. (1962). The norm of the Schur product operation. Numer. Math..

[36] Srivastava N., Hinton G., Krizhevsky A., Sutskever I., Salakhutdinov R. (2014). Dropout: A Simple Way to Prevent Neural Networks from Overfitting. J. Mach. Learn. Res..

[37] Kingma D.P., Ba J. Adam: A Method for Stochastic Optimization. Proceedings of the 3rd International Conference for Learning Representations.

[38] Chen M., AlRegib G., Juang B.H. (2013). Feature processing and modeling for 6D motion gesture recognition. IEEE Trans. Multimed..

[39] Yana B., Onoye T. (2018). Fusion networks for air-writing recognition. Lecture Notes in Computer Science (Including Subseries Lecture Notes in Artificial Intelligence and Lecture Notes in Bioinformatics).

